# Hydroa vacciniforme-like lymphoproliferative disorder (HV-LPD) is an Epstein-Barr virus (EBV) associated disease^☆^^☆☆^

**DOI:** 10.1016/j.abd.2020.06.023

**Published:** 2021-03-24

**Authors:** Juliana Ordoñez-Parra, Maddy Mejía Cortes, Maria Margarita Tamayo-Buendía, Ana María Infante Gómez

**Affiliations:** Hospital Universitario San Ignacio, Bogotá, Colombia

Dear Editor,

We present a 12-year-old Hispanic male with a 6-year history of “nodules” that ulcerated in the face, lower and upper limbs which left multiple scars. He attended for 20-days of facial edema, associated with a decrease in visual acuity. The ophthalmologist reported necrosis of the left eye. During the physical examination he presented periorbital edema, left frontal vesico-blisters, which left varioliform scars (Fig. 1). A skin biopsy was performed with evidence of epidermal necrosis, atypical perivascular, and perianexial lymphoid infiltrates with angiocentricity. Immunohistochemistry was compatible with cytotoxic T lymphocytes (CD3^+^ CD8^+^ Perforine^+^ CD56-) with a 20% of ki67, and a positive in situ hybridization for Epstein-Barr virus (EBER) test (Figure 2, Figure 3). A conjunctiva biopsy was performed, with evidence of necrotic tissue, and a positive polymerase chain reaction for EBV. Viral load for EBV in blood was positive (197,929 copies/mL). With all of the above, a diagnosis of HV-LPD was performed. CT scans report cervical adenopathies and hepatosplenomegaly. Biopsy of cervical node and bone marrow was negative for malignancy. Proper treatment with oral thalidomide 100 mg QD was initiated, achieving clinical response.

**Figure 1:**
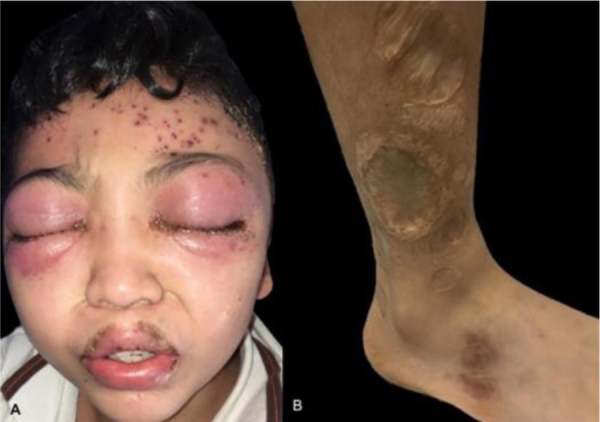
(A), Periorbital edema, and erythema with left frontal vesico-blisters with hemorrhagic content. (B), Multiple atrophic and some anetodermic scars on the lower limbs.

**Figure 2:**
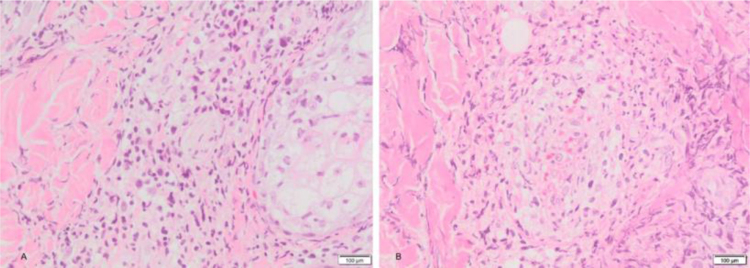
(A), Atypical perivascular and perianexial lymphoid infiltrates. (B), Angiocentricity.

**Figure 3:**
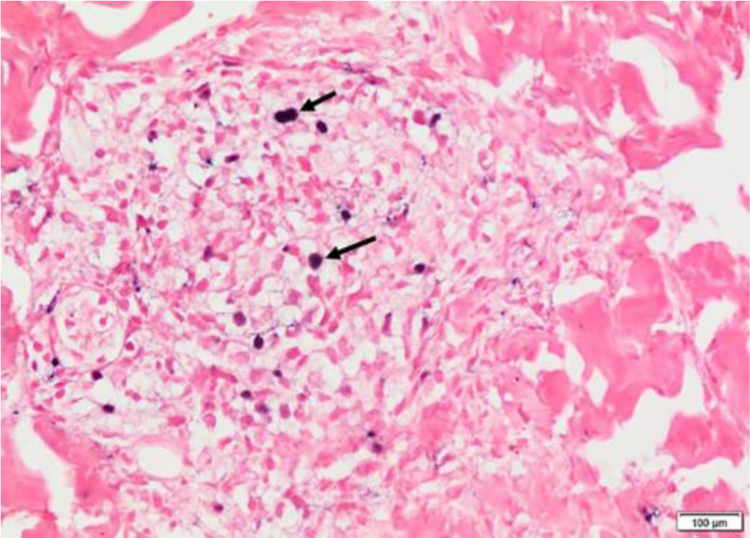
Positive in-situ hybridization for EBV (black arrows) (Hematoxylin & eosin, ×100).

American cases of HV-LPD have been described in children of Mexico, Peru, and Bolivia.^1^, ^2^ According to some studies viral DNA is elevated in most patients, suggesting a chronic EBV infection and a genetic susceptibility for defective EBV-specific immunity.^3^, ^4^ Clinically, they present as papulovesicular eruptions, with necrotic centers, in sun-exposed and non-exposed areas.^1^ Lie et al. report 12% of patients with ocular symptoms, including corneal nebula, conjunctival swelling, photophobia, and tearing.^5^ Unfortunately, our patient had severe ocular involvement which, in the best of our knowledge, there are no previous reports on such association. Therefore, we propose this new feature, due to the clinical picture and positivity for EBV DNA.

Histologically, the epidermis displays extensive ulceration with necrosis and angiocentricity as a common finding in the vessels.^1^ Frequent findings include a dense infiltrate of small-to middle size atypical lymphoid cell, pleomorphic nuclei, mainly located around adnexae and blood vessels.^1^, ^4^

Immunohistochemistry reported a lymphoid population (CD3^+^, CD5^+^, and CD7^+^)^1^ with cytotoxic or natural killer phenotype (CD8^+^ and CD56^+^ respectively).^1^ The most frequent phenotype is a cytotoxic lymphoid infiltrate, like in our patient, with positive cytotoxic markers.^1^, ^2^, ^3^ Performance of EBER’s is encoded for the detection of EBV RNA, positivity of this test have been report up to 100%.^1^, ^3^, ^5^

The main differential diagnoses are classic hydroa vacciniforme. As HV-LPD, classic hydroa vacciniforme appears in children, has a papulovesicular eruption with a crusted center, and posterior varioliforme scarring. Nonetheless, its localization is limited to sun-exposed areas, with no facial edema nor systemic compromise. Histologic differential diagnosis includes other lymphomas associated with EBV. For example, nasal NK lymphoma is characterized by a rapidly progressive clinical course, positive CD56 and negative CD8 on the immunohistochemistry.

There are no treatment guidelines due to the rarity of the disease. Beltran et al. consider thalidomide as a useful treatment, due to its anti-inflammatory and antiproliferative properties.^2^ Four Peruvian patients received thalidomide 100 mg orally daily with different results.^2^ We report a 12-month clinical remission in our patient, following thalidomide treatment, supporting the use of it as a first-line immunomodulating agent.

## Financial support

None declared.

## Authors’ contributions

Juliana Ordoñez-Parra: Patient assessment; literature review; manuscript creation.

Maddy Mejía Cortes: Patient assessment; literature review; manuscript creation.

Margarita Tamayo-Buendía: Patient assessment; literature review; manuscript creation.

Ana María Infante Gómez: Patient assessment and literature review.

## Conflicts of interest

None declared.
